# Immunohistological Analysis of Neutrophils and Neutrophil Extracellular Traps in Human Thrombemboli Causing Acute Ischemic Stroke

**DOI:** 10.3390/ijms21197387

**Published:** 2020-10-07

**Authors:** Fabian Essig, Alexander M. Kollikowski, Mirko Pham, László Solymosi, Guido Stoll, Karl Georg Haeusler, Peter Kraft, Michael K. Schuhmann

**Affiliations:** 1 Department of Neurology, University Hospital Würzburg, 97080 Würzburg, Germany; Essig_F@ukw.de (F.E.); Stoll_G@ukw.de (G.S.); Haeusler_K@ukw.de (K.G.H.); Peter.kraft@klinikum-msp.de (P.K.); 2 Department of Neuroradiology, University Hospital Würzburg, 97080 Würzburg, Germany; Kollikowsk_A@ukw.de (A.M.K.); Pham_M@ukw.de (M.P.); Solymosi_L@ukw.de (L.S.); 3 Department of Neurology, Klinikum Main-Spessart, 97816 Lohr, Germany

**Keywords:** acute ischemic stroke, thrombemboli, neutrophils, NETs, immunohistochemistry

## Abstract

Ischemic stroke caused by thromboembolic occlusion of large cerebral arteries, such as the internal carotid (ICA) and/or the middle cerebral artery (MCA), is treated by mechanical thrombectomy (MT). MT allows salvage of the vessel-occluding thrombemboli, which most frequently originate from the left atrium or the left ventricle of the heart or from sites of plaque rupture within large arteries above the heart. Clot composition may influence the efficacy of (intravenous) thrombolysis and MT, respectively. We analyzed 37 human thrombemboli obtained from acute ischemic stroke patients during MT with special emphasis on histological staining of neutrophils and neutrophil extracellular traps (NETs). We found neutrophils as the main cellular component of cerebral thrombemboli but encountered considerable morphological heterogeneity. Neutrophils accumulated in the border region of fibrin-rich structures indicating possible interaction of neutrophils with distinct structural thrombembolus components. Web-like NETs were found in 35 of 37 thrombemboli in varying amounts. NETs were almost exclusively found within fibrin-rich areas. Importantly, stroke etiology, age and present oral anticoagulation was associated with morphological patterns and the amount of neutrophils. Correlation of histological data and imaging data revealed that relative Hounsfield units of cerebral thrombemboli positively correlated with the amount of red blood cells. In summary, our results demonstrate that neutrophils and NETs are substantial constituents of cerebral thrombemboli and contribute to their structural complexity.

## 1. Introduction

The primary goal of acute stroke treatment is rapid recanalization by either intravenous thrombolysis (IVT) using recombinant tissue plasminogen activator (rt-PA) and/or mechanical thrombectomy (MT) if large intracranial arteries or arterial segments are occluded [1]. With the advent of MT, occlusive thrombemboli have become obtainable for detailed histological analysis, thereby providing insights into the composition and structural organization of the occluding clots, potentially also of their embolic origin as well as mechanisms of thrombus formation and in situ reorganization. So far, numerous studies have investigated the histopathological composition of ischemic stroke thrombemboli and identified red blood cells (RBCs), fibrin, platelets, von Willebrand Factor (vWF) and white blood cells as main components [2,3,4,5,6]. While immunohistological analysis of lymphocytes and monocytes was frequently performed, only a few studies focused on neutrophils and neutrophil extracellular traps (NETs) [7,8]. NETs are web-like structures of deoxyribonucleic acid (DNA) fibers comprising histone and granules that are released by activated neutrophils. NETs are thought to be actively involved in thrombosis by interacting with platelets, RBCs and platelet adhesion molecules such as fibrinogen, vWF and fibronectin [9,10]. High numbers of neutrophils were shown in cerebral thrombemboli and a contribution of the release of NETs to potentially impaired efficacy of IVT was discussed [11,12]. Subsequently, detailed knowledge of the complex and variable histopathologic characteristics of thrombemboli might be relevant to guide future advancements in acute ischemic stroke (AIS) treatment [13]. With these considerations in mind, we investigated the cellular and structural organization of large vessel-occluding cerebral thrombemboli with a focus on neutrophils and NETs. Furthermore, we correlated immunohistological findings with clinical characteristics and imaging parameters.

## 2. Results

### 2.1. Characterization of Thrombemboli

**Figure 1:**
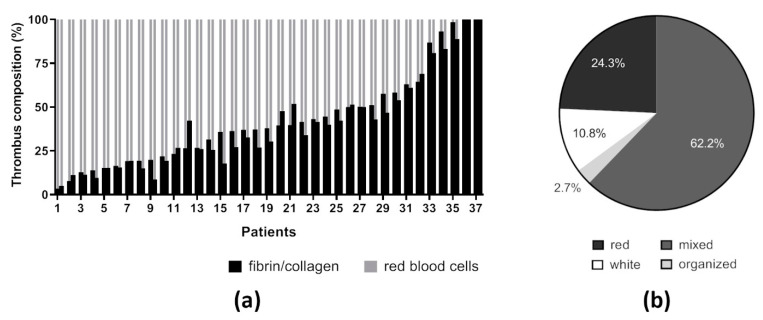
Characterization of thrombemboli. (**a**) Classification of occlusive thrombemboli due to their two main components: red blood cell-rich and fibrin/collagen-rich areas in percentage of total area for two different thrombemboli slices represented by two separate bars per patient. (**b**) Categorization of thrombemboli based on quantitative morphological area analysis.

Thrombemboli were retrieved by MT in 37 AIS patients with large vessel occlusion and analyzed immunohistologically. The median age of patients was 63 (56–77) years and the gender of patients was equally distributed. Before MT, 70% of patients received IVT (Table 1). Based on histological examination, thrombemboli were classified according to their main components: RBC and fibrin/collagen. Quantitative analysis revealed great heterogeneity within retrieved thrombemboli with varying amounts of RBC- and fibrin/collagen- dominant areas. By comparing two different slices of a single thrombembolus, we demonstrate similar composition within identical thrombemboli (Figure 1). Overall, the amount of RBC-rich areas ranged from 0 to 96.7% (mean 57.6%) and for fibrin/collagen areas from 3.3 to 100% (mean 42.4%) (Figure 1a). According to their main structural components, thrombemboli were assigned as red (24.3%), white (10.8%), organized (2.7%) or mixed (62.2%) (Figure 1b).

### 2.2. Neutrophils Are Abundant in Cerebral Thrombemboli

To assess the role of neutrophils in ischemic stroke thrombemboli derived from large vessel occlusion we stained for CD66b and neutrophil elastase (NE). Neutrophils were abundant in all 37 thrombemboli with 703.3 ± 424.2 cells/mm^2^ (Supplement Table S1), and the number of neutrophils was associated with stroke severity as assessed by the National Institutes of Health Stroke Scale (NIHSS) on admission (*r* = 0.27; *p* = 0.09) and dismissal (*r* = 0.29, *p* = 0.07) (Supplement Table S1). To exclude that neutrophil accumulation in thrombemboli was only due to raised blood leukocyte counts, we performed linear regression analysis, revealing no correlation between blood leukocyte count and the amount of neutrophils in cerebral thrombemboli (*r* = 0.0005, Supplement Figure S1). 

**Figure 2:**
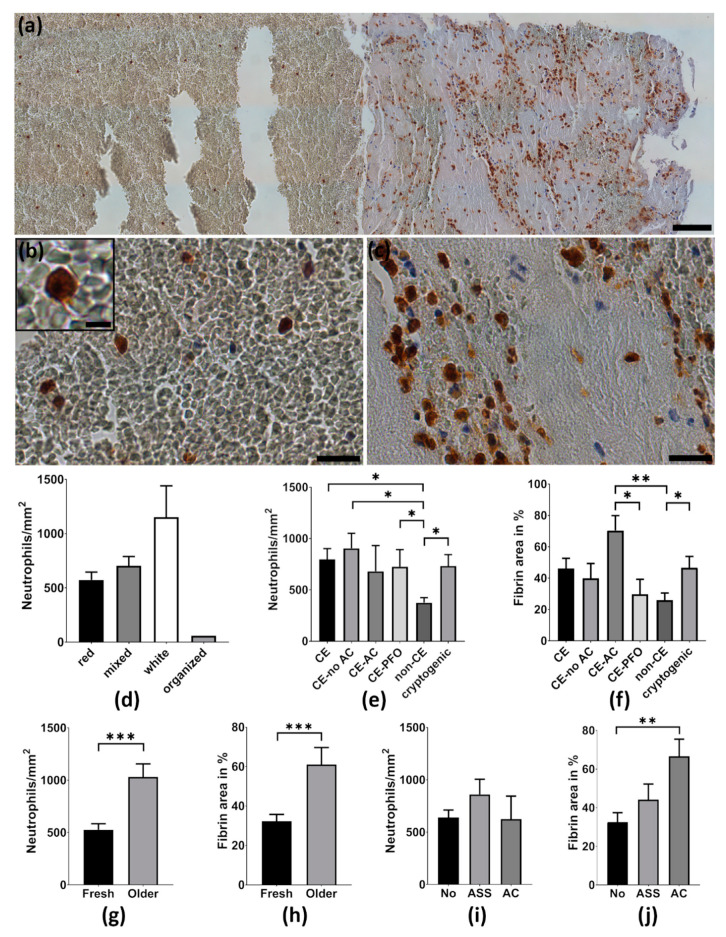
Neutrophils and structural composition in thrombemboli. (**a**) Representative image of an anti-CD66b stained thrombemboli displaying histopathological heterogeneity. Nuclear cells (dark blue) are counterstained with hemalum staining. (**b**) Magnifications of red blood cell (RBC)-dominant area with additional magnification (scale bar 10 µm) of a neutrophil in the upper left corner and (**c**) fibrin-dominant area illustrating specific structural accumulation behavior of neutrophils. (**d**–**j**) Quantitative analysis of neutrophils and fibrin area regarding (**d**) thrombemboli morphology, (**e**,**f**) stroke etiology, (**g**,**h**) thrombembolus age and (**i**,**j**) patients’ premedication without anticoagulant therapy (No), with acetylsalicylic acid (ASA) or with pre-existing anticoagulation (AC). CE = cardioembolic, CE-no AC = cardioembolic without anticoagulation, CE-AC = cardioembolic with anticoagulation, CE-PFO = cardioembolic with patent foramen ovale, non-CE = non-cardioembolic. * *p* < 0.05, ** *p* < 0.01, *** *p* < 0.001. Scale bars: (**a**) 100 µm, (**b**,**c**) 20 µm.

The number of neutrophils per mm^2^ differed strongly between thrombemboli with a lowest value of 58.1 cells/mm^2^ and the highest neutrophil amount of 1684.5 cells/mm^2^. Immunohistological analysis revealed that neutrophils were not spread evenly within thrombemboli (Figure 2a). Whereas in fibrin-rich areas neutrophils accumulated significantly and seemed to encircle fibrin-rich structures (Figure 2c), neutrophils were rare and homogeneously distributed within RBC-dominant areas (Figure 2b). Total quantitative analysis of neutrophils within thrombemboli revealed that RBC-rich thrombemboli (median 573.5 cells/mm^2^) contain numerically less neutrophils than fibrin/collagen-rich thrombemboli (median 1152 cells/mm^2^, *p* = 0.1). No differences were found for mixed thrombemboli (median 704 cells/mm^2^) compared to RBC-rich or fibrin/collagen-rich thrombemboli (Figure 2d).

### 2.3. Thrombemboli Composition and Stroke Etiology

For all patients Trial of Org 10172 in Acute Stroke Treatment (TOAST) classification was used to determine stroke etiology as cardioembolic (atrial fibrillation, aortic valve replacement, dilated cardiomyopathy; *n* = 21), non-cardioembolic (large artery atherosclerosis, carotid dissection, *n* = 7) and cryptogenic (*n* = 9). Analysis revealed that neutrophil numbers were higher in thrombemboli of presumed cardioembolic origin (799.1 ± 477.6 cells/mm^2^) and in thrombemboli of cryptogenic origin (734.1 ± 329.1 cells/mm^2^) compared to non-cardioembolic thrombemboli (376 ± 128.5 cells/mm^2^, *p* = 0.03, *p* = 0.02). Comparing “cardioembolic” stroke patients without pre-existing anticoagulation and with patent foramen ovale (PFO) revealed higher neutrophil numbers in stroke patients without pre-existing medical anticoagulation (905.8 ± 460.7 cells/mm^2^, *p* = 0.01) and also for cardioembolic stroke due to PFO (726.5 ± 370.6 cells/mm^2^, *p* = 0.04) compared to non-cardioembolic stroke etiology (Figure 2e). 

The fibrin area of thrombemboli due to cardioembolic stroke with pre-existing anticoagulation (70.3 ± 23.5 %) was higher than in cardioembolic thrombemboli due to PFO (29.7± 21.4%, *p* = 0.02) and non-cardioembolic thrombemboli (25.9 ± 12.1%, *p* = 0.001). Cerebral thrombemboli of cryptogenic origin had a higher proportion of fibrin (46.6 ± 21.8%) than non-cardioembolic thrombemboli (25.9 ± 12.1%, *p* < 0.04) and similar fibrin content in comparison to cardioembolic cerebral thrombemboli (46.1 ± 29.9%) (Figure 2f). Additionally, we determined the age of thrombemboli as fresh (<1 day) and older (>1 day), which revealed that older thrombemboli contain more neutrophils than fresh thrombemboli (*p* = 0.0002) and their fibrin area is also higher than in fresh thrombemboli (*p* = 0.0008) (Figure 2g,h).

### 2.4. Thrombemboli Composition and Medication at Stroke Onset

Thrombemboli composition according to premedication with acetylsalicylic acid (ASA), oral anticoagulation or no antiplatelet therapy was compared. Interestingly, the proportion of fibrin was higher in patients with pre-existing anticoagulation (66.6 ± 23.5%) than in patients without anticoagulant therapy (32.5 ± 21.5%, *p* = 0.002). No differences were found for patients with ASA (44.2 ± 27%, *p* = 0.09) (Figure 2j). Likewise, no differences in the number of neutrophils were observed in patients without anticoagulant therapy, ASA or with pre-existing anticoagulation (Figure 2i).

### 2.5. Neutrophil Extracellular Traps in Cerebral Thrombemboli

**Figure 3:**
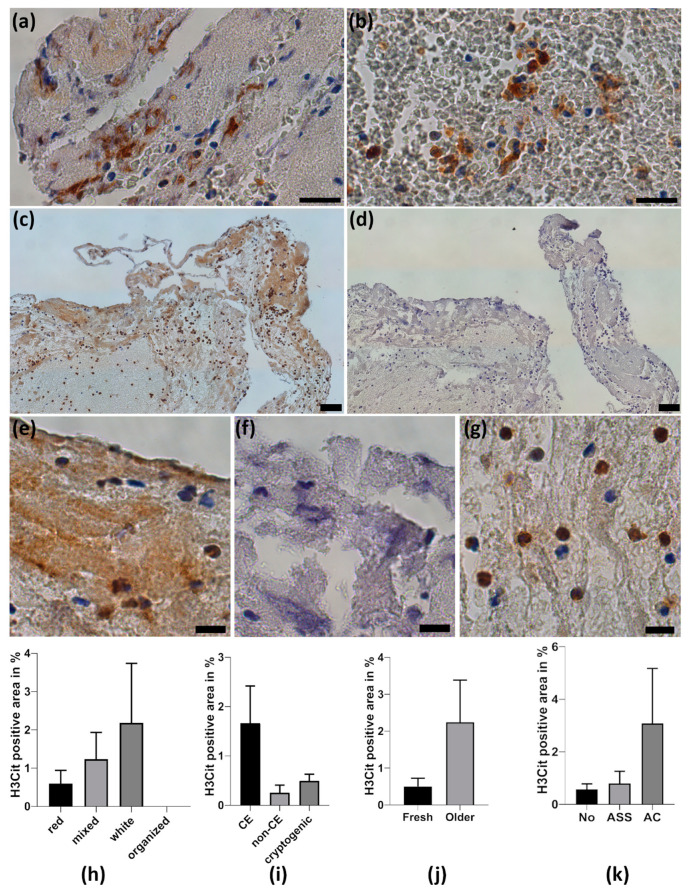
Neutrophil extracellular traps in thrombemboli. (**a**,**b**) Representative H3Cit stained thrombemboli. (**a**) Magnifications of web-like neutrophil extracellular traps (NETs) within fibrin-rich structures and (**b**) web-like NETs in RBC-dominating area. (**c**) Cerebral thrombembolus with a vast amount of H3Cit-positive area and control staining with IgG (**d**). (**e**) Magnification illustrates the presence of H3Cit-positive area identified by its brown color in contrast to IgG control (**f**) and intracellular H3Cit immunoreactivity in neutrophils (**g**). Counterstaining with hemalum dyes nuclear cells and DNA. H3Cit-positive area was quantified and is presented according to thrombembolus morphology (**h**), stroke etiology (**i**), thrombembolus age (**j**) and pre-existing anticoagulant medication (**k**). ASA = acetylsalicylic acid, AC = anticoagulation. Scale bars: (**a**,**b**) 20 µm, (**c**,**d**) 50 µm; (**e**,**f**,**g**) 10 µm.

Quantitative analysis of NETs by H3Cit staining revealed that web-like NETs were found in 35 of 37 thrombemboli, accounting for 1.1 ± 2.7% of total thrombemboli area (Supplement Table S1). Web-like NETs were mainly found within fibrin-rich structures (Figure 3a), whereas web-like NETs in RBC-rich areas were a rare finding (Figure 3b). We detected intracellular H3Cit positive neutrophils in all thrombemboli in varying amounts (Figure 3g). Only a minority of thrombemboli contain high amounts of H3Cit-positive area up to 14.3% of total thrombembolus area (Figure 3c–f). 

NETs were more often found in white thrombemboli than in red or mixed thrombemboli. They were more common in presumed cardioembolic thrombemboli than in non-cardioembolic or cryptogenic thrombemboli, were found more frequently in older than in fresh thrombemboli and were detected more often in thrombemboli from patients with pre-existing anticoagulation than in patients who received ASA or no anticoagulant medication (Figure 3h–k).

**Figure 4:**
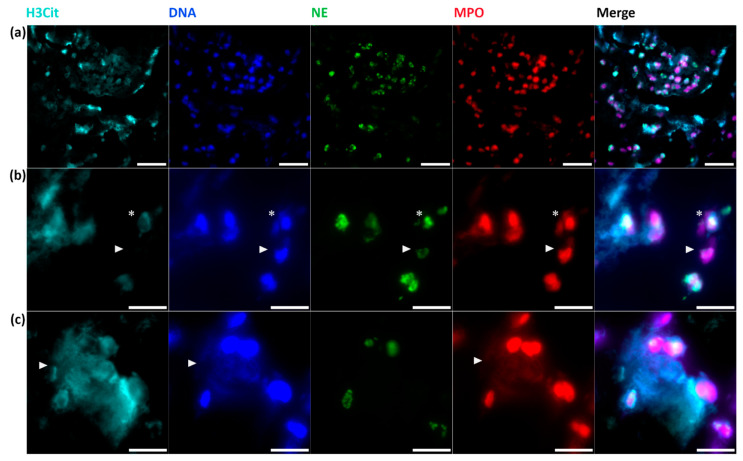
Immunofluorescent staining of NETs. Immunofluorescent co-staining identifies (**a**) large thrombembolus area with co-localization of H3Cit, DNA (by DAPI), NE and MPO. Representative magnifications of (**b**) NETosis (*) and neutrophils without intracellular H3Cit *(arrowhead)* and (**c**) web-like NETs with co-localization of extracellular H3Cit, DNA and MPO are shown *(arrowhead)*. Scale bars: (**a**) 20 µm, (**b**,**c**) 10 µm. NE = neutrophil elastase; MPO = myeloperoxidase, DAPI = 4,6-diamidino-2-phenylindole.

In addition, immunofluorescent co-staining of H3Cit, NE, myeloperoxidase (MPO) and a DNA dye (DAPI= 4,6-diamidino-2-phenylindole) was performed to visualize NETs (Figure 4). In line with the immunohistochemical results, fluorescent staining confirms the presence of neutrophils without intracellular H3Cit, intracellular H3Cit-positive neutrophils called “NETosis” (Figure 4b) and further demonstrated that web-like NETs consist of extracellular H3Cit, DNA and MPO originating from neutrophils, which was proved by the close spatial proximity of NE (Figure 4c).

### 2.6. Thrombembolus Histopathology and Imaging Findings

**Figure 5:**
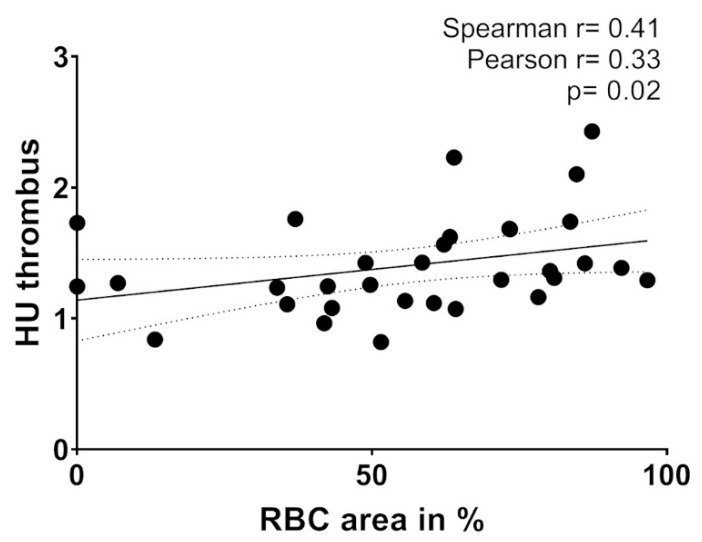
Correlation between the area of red blood cells (RBCs) (in % of total thrombembolus area) and relative HU (Hounsfield unit) density on non-contrast CT (NCCT). *p* = level of significance. *r* = correlation coefficient.

Correlation analysis with predefined non-invasive imaging and interventional parameters was performed to assess the impact of histopathology on the efficacy of recanalization therapy. Parameters included vessel occlusion location, baseline Alberta Stroke Program Early CT Score (ASPECTS) values, relative Hounsfield units (rHU) [14], duration of MT procedure and the number of stent retriever maneuvers. A positive correlation between the area of RBCs (in % of total thrombembolus area) and relative HU density on non-contrast CT (NCCT) was observed (*r* = 0.41, *p* = 0.02, *n* = 32) (Figure 5). No correlation of non-invasive imaging or interventional parameters was found for neutrophils, NETs and the amount of fibrin (Supplementary Table S1).

## 3. Discussion

Immunohistological analysis of human thrombemboli causing AIS became available since mechanical recanalization was established [13]. In contrast to previous studies focusing on morphological hallmarks, neutrophil numbers and NETs in cerebral thrombemboli, we performed a quantitative analysis correlating histological findings with clinical and brain imaging features.

As principal finding, we detected a morphological heterogeneity in thrombembolus composition and found neutrophils as the most abundant cellular infiltrate within thrombemboli. Importantly, in fibrin-rich structures, neutrophils seem not only to accumulate but also to encircle the fibrin-rich structures, indicating an interaction between immune cells and fibrin. Our results correspond to recent work that found leukocyte accumulation in platelet-rich areas of human thrombemboli consisting of a meshwork of thin fibrin with vWF and DNA [13,15]. Notably, additional ADAMTS13 and DNAse application improved the degradation of cerebral thrombus material [11,12,16]. 

Our data confirm the occurrence of web-like NETs throughout the majority of thrombemboli, whereas NETs can be found almost exclusively within fibrin-rich areas. NETs are thought to contribute to structural stabilization, and, in fact, the addition of histone-DNA complexes to fibrin resulted in thicker fibers accompanied by increased rigidity [17]. NET formation was detected inside blood vessels and cerebral parenchyma in the peri-infarct cortical areas impairing revascularization and vascular remodeling after stroke [18]. Recently, NETs were detected more often in thrombemboli retrieved from patients with AIS than in acute myocardial infarction. NET abundance was associated with poor outcome in patients with AIS and with reduced ejection fraction in patients with acute myocardial infarction [19]. These findings underline the importance of NETs in thrombemboli and further point towards major differences concerning thrombus formation between various ischemic vascular events. 

Based on the huge morphological heterogeneity of thrombemboli it is conceivable that thrombus formation is heterogeneous, as demonstrated in our study. Our data reveal major differences in total amount of neutrophils and thrombembolus composition for cardioembolic, non-cardioembolic and cryptogenic stroke etiology. Moreover, our study suggests differences according to subcategorization of cardioembolic stroke and patients’ anticoagulant premedication: Fibrin content of thrombemboli suggested due to PFO was found to be low, which is in accordance with previous data for venous thrombemboli with predominant components of entrapped RBC [6], while thrombemboli from patients with anticoagulant premedication contained high amounts of fibrin. Additionally, we found a positive association of the thrombembolus RBC content with rHU on pre-interventional NCCT adjusting for hematocrit. In accordance with our results, numbers of leukocytes and proportions of fibrin/platelets were found higher in patients with presumed cardioembolic stroke in contrast to non-cardioembolic thrombemboli [20,21]. Detailed knowledge about thrombembolus composition regarding thrombembolus morphology and stroke etiology might thereby help to determine stroke origin and improve strategies for secondary stroke prevention. Furthermore, emerging evidence indicates that RBC-rich thrombemboli might be more accessible for intravenous lysis therapy in contrast to fibrin-rich thrombemboli [22]. RBC-rich thrombemboli were shown to be associated with reduced numbers of stent retriever maneuvers and successful thrombectomy but also as independent predictor for clot migration, which might have a negative impact on the technical and clinical outcome [23,24]. 

Taken together, our results reveal that beside simplified morphological categorization of cerebral thrombemboli, interaction of neutrophils and NETs with structural thrombembolus components as well as thrombemboli origin and anticoagulant premedication elucidate the complexity of thrombus formation, which may result in additional structural stability and cause thrombolytic resistance.

## 4. Materials and Methods

### 4.1. Patient Population and Study Design

We histologically analyzed 37 human thrombemboli causing AIS that were retrieved during MT at the Department of Neuroradiology, University Hospital of Würzburg, Germany as previously described [7]. The study protocol was approved by the ethics committee of the Medical Faculty of the University of Würzburg, Germany (reference number 36/12, 13 March 2012), and written informed consent was provided by all participants. Cerebral thrombemboli were taken from patients >18 years with large vessel occlusion of the middle cerebral artery (MCA), internal carotid artery (ICA) or basilar artery when MT was successful and there was informed consents of the patient or their legal representatives during hospital treatment. Clinical parameters such as NIHSS score, TOAST (Trial of Org 10172 in Acute Stroke Treatment) classification, treatment with rt-PA or stroke etiology were collected from patient data.

### 4.2. Thrombectomy Procedure and Neuroradiological Analysis

MT was performed using stent retrievers according to local standard. Patients were treated under general anesthesia during mechanical recanalization. Imaging data comprised vessel occlusion location on CT angiography, relative Hounsfield Units (rHU) of the thrombembolus [14], the Alberta Stroke Program Early CT score (ASPECTS) on baseline imaging, recanalization time and the number of stent retriever maneuvers. 

### 4.3. Processing of Thrombemboli and Immunohistochemistry for Neutrophils and NETs

Initial thrombembolus processing was performed as described previously [7]. In brief, after retrieval thrombembolus material was fixed in phosphate-buffered formalin, embedded in paraffin (Leica, Wetzlar, Germany) and cut into 4-μm sections. 

Thrombemboli were stained for hematoxylin and eosin (H&E), CD66b, NE and H3Cit. The fixed thrombembolus slices were dewaxed in Xylol for 20 min and blocked with peroxidase for 15 min. Thrombembolus sections were washed in Tris-buffered saline containing 0.1% Tween 20. Sections were blocked for 1 h in a buffer containing (10% serum, 1% bovine serum albumin (BSA) and 0.1% Tween 20). Primary antibodies (rabbit antihuman histone H3 (citrulline R218117, H3Cit) polyclonal antibody (1:50, ab5103; Abcam, Cambridge, UK), mouse anti-human neutrophil elastase (NE) monoclonal antibody (1:100, M0752; Dako, Glostrup, Denmark) and a rabbit anti-human CD66b polyclonal antibody (1:50, Ab197678, Abcam)) were incubated overnight at 4 °C. After three times washing, a biotinylated secondary anti-rabbit IgG antibody (1:100, BA-1000, Vector Laboratories Peterborough, United Kingdom) or biotinylated anti-mouse IgG antibody (1:100, BA-9200, Vector Laboratories) was incubated for 45 min. Avidin-biotinylated enzyme complex (VECTASTAIN ABC kit; Vector Laboratories) followed by diaminobenzidine (K3467, Dako) was used to detect biotinylated antibodies and visualize H3Cit, CD66b or NE. DNA and nuclear cells were visualized by counterstaining with hemalum. As negative controls, isotype primary antibodies (H3Cit and CD66b: polyclonal rabbit IgG isotype control antibody) (910801, Biolegend); NE: monoclonal mouse IgG1k isotype control antibody (401402, Biolegend) were used. Leica DMi8 and LasX software was used for image acquisition.

As both staining methods for CD66b and NE were suitable to identify neutrophils, CD66b staining was used for further analysis. Quantification of neutrophils was performed manually by counting all CD66b positive cells within one slide. The area of thrombemboli in mm^2^ (total thrombembolus area, erythrocyte-dominant area, fibrin-dominant area and H3Cit-positive area) was determined by Image J software (https://imagej.nih.gov/ij/). According to their main structural components, thrombemboli were assigned to four different subtypes. Thrombemboli were classified as red (RBCs outnumber fibrin/collagen ≥80%), white (fibrin/collagen outnumber RBCs ≥80%), mixed (RBC-fibrin/collagen area >20% and <80%) and organized (organized collagen structure). Thrombembolus age was classified according to established histopathological definitions by two independent persons [11].

### 4.4. Immunofluorescence Staining of NETs

For immunofluorescence staining, thrombembolus sections were dewaxed, blocked with 5% BSA and 0.2% Triton in PBS for one hour. Primary antibodies (rabbit anti-human histone H3 (citrulline R218117, H3Cit) polyclonal antibody (1:50, ab5103; Abcam, Cambridge, UK), mouse anti-human neutrophil elastase (NE) monoclonal antibody (1:100, M0752; Dako, Glostrup, Denmark) and goat anti-human MPO polyclonal antibody (1:100, AF 3667, R&D Systems, Minneapolis, MN, USA)) were incubated overnight at 4 °C. After washing, secondary antibodies (Alexa Fluor 488 goat anti-mouse IgG) (1:100, A11001, Invitrogen, Carlsbad, USA), Alexa Fluor 647 goat anti-rabbit IgG (1:100 A21244, Invitrogen) and Cy3 donkey anti-goat IgG (1:200 705-165-147, Dianova, Hamburg, Germany) were incubated for one hour at room temperature. Slices were counterstained with ProLong Gold Antifade Mountant with DAPI (P36931, Invitrogen). For negative controls, primary antibodies were not used. Leica DMi8 and LasX software was used for image acquisition.

### 4.5. Statistical Analysis

Data are presented as mean values ± SEM of the mean. Statistical analysis was performed with Graph Pad Prism 8.0.2 (Graph Pad Software, San Diego, CA, USA). Normal distribution of the datasets was tested using the Kolmogorov–Smirnov normality test. A T-test, the Mann–Whitney U test and the Pearson/Spearman correlation were performed to test for significance. Two-sided *p* values <0.05 were considered to indicate statistical significance.

## Figures and Tables

**Table 1:** Clinical characteristics of patients and collected thrombemboli for histological analysis.

Characteristics	Value
Age, year, median (IQR)	63 (56–77)
Gender, No. (%)	
Male	18 (48.6)
Female	19 (51.4)
Stroke etiology, No. (%)	
Cardioembolic	21 (56.8)
CE-no AC	10 (27.0)
CE-AC	6 (16.2)
CE-PFO	5 (13.5)
non-cardioembolic	7 (18.9)
cryptogenic	9 (24.3)
IV rt-PA, No. (%)	
Yes	26 (70.3)
No	11 (29.7)
NIHSS, median (IQR)	
Admission	17 (13–22)
Dismissal	8 (4–10)

Abbreviations: CE = cardioembolic; AC = anticoagulation; PFO = patent foramen ovale, IV rt-PA = intravenous recombinant tissue type plasminogen activator, NIHSS = National Institutes of Health Stroke Scale.

## Supplementary Materials

Supplementary materials can be found at https://www.mdpi.com/1422-0067/21/19/7387/s1. Table S1: Correlation of histologic results with clinical and imaging findings; Figure S1: Correlation of blood leukocytes with neutrophils in thrombemboli.

## Abbreviations

AIS	acute ischemic stroke
ASPECTS	Alberta Stroke Program Early CT Score
ASA	acetylsalicylic acid
CD	cluster of differentiation
DNA	deoxyribonucleic acid
DAPI	4,6-diamidino-2-phenylindole
H&E	hematoxylin and eosin
HU	Hounsfield unit
ICA	internal carotid artery
IVT	intravenous thrombolysis
MT	mechanical thrombectomy
MCA	middle cerebral artery
MPO	myeloperoxidase
NCCT	non-contrast computer tomography
NIHSS	National Institutes of Health Stroke Scale
NE	neutrophil elastase
NET	neutrophil extracellular traps
PFO	patent foramen ovale
RBC	red blood cell
rt-PA	recombinant tissue plasminogen activator
SEM	standard error of the mean
TOAST	Trial of Org 10172 in Acute Stroke Treatment
vWF	von Willebrand Factor
