# Supplementary material for: Immunohistological Analysis of Neutrophils and Neutrophil Extracellular Traps in Human Thrombemboli Causing Acute Ischemic Stroke
Source: Int J Mol Sci. 2020 Oct 7;21(19):7387. doi: 10.3390/ijms21197387 (PMC7582743; doi:10.3390/ijms21197387)
Supplement: Supplementary file 1 [file ijms-21-07387-s001.zip › Supplement Figure 1.pdf]

Immunohistological analysis of neutrophils and neutrophil extracellular traps in human thrombemboli causing acute ischemic stroke

Fabian Essig[1], Alexander M. Kollikowski[2], Mirko Pham[2], László Solymosi[2], Guido Stoll[1], Karl Georg Haeusler[1], Peter Kraft[1,3], Michael K. Schuhmann[1,*]

[1] Department of Neurology, University Hospital Würzburg, 97080 Würzburg, Germany; Essig_F@ukw.de (F.E), Stoll_G@ukw.de (G.S), Haeusler_K@ukw.de (K.G.H), Schuhmann_M@ukw.de (M.K.S)
[2] Department of Neuroradiology, University Hospital Würzburg, 97080 Würzburg, Germany; Kollikowsk_A@ukw.de (A.M.K), Pham_M@ukw.de (M.P), Solymosi_L@ukw.de (L.S)
[3] Department of Neurology, Klinikum Main-Spessart, 97816 Lohr, Germany; Peter.kraft@klinikum-msp.de
* Correspondence: Schuhmann_M@ukw.de; Tel.: +49 931 201 23653

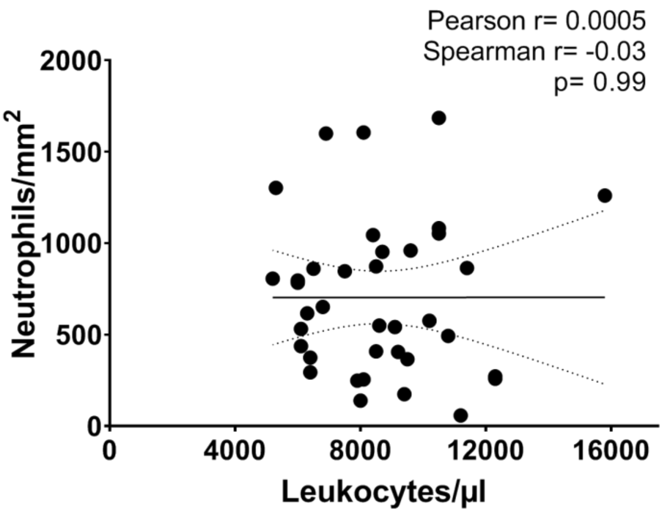

Supplement Figure 1. Correlation between the blood leukocytes/µl and the number of neutrophils/mm^2. p = level of significance. R = correlation coefficient.
