# Supplementary material for: Immunohistological Analysis of Neutrophils and Neutrophil Extracellular Traps in Human Thrombemboli Causing Acute Ischemic Stroke
Source: Int J Mol Sci. 2020 Oct 7;21(19):7387. doi: 10.3390/ijms21197387 (PMC7582743; doi:10.3390/ijms21197387)
Supplement: Supplementary file 1 [file ijms-21-07387-s001.zip › Supplement Table 1.pdf]

Immunohistological analysis of neutrophils and neutrophil extracellular traps in human thrombemboli causing acute ischemic stroke

Fabian Essig[1], Alexander M. Kollikowski[2], Mirko Pham[2], László Solymosi[2], Guido Stoll[1], Karl Georg Haeusler[1], Peter Kraft[1,3], Michael K. Schuhmann[1,*]

[1] Department of Neurology, University Hospital Würzburg, 97080 Würzburg, Germany; Essig_F@ukw.de (F.E), Stoll_G@ukw.de (G.S), Haeusler_K@ukw.de (K.G.H), Schuhmann_M@ukw.de (M.K.S)
[2] Department of Neuroradiology, University Hospital Würzburg, 97080 Würzburg, Germany; Kollikowsk_A@ukw.de (A.M.K), Pham_M@ukw.de (M.P), Solymosi_L@ukw.de (L.S)
[3] Department of Neurology, Klinikum Main-Spessart, 97816 Lohr, Germany; Peter.kraft@klinikum-msp.de
* Correspondence: Schuhmann_M@ukw.de; Tel.: +49 931 201 23653

Supplement Table 1. Correlation of histologic results with clinical and imaging findings.

	Neutrophils (n/mm2)	NETs (in %)	Fibrin Area (in %)
Thrombemboli (n=37)	703.3 ± 424.2	1.11 ± 2.7	43.1 ± 26.6
ICA-T (n=13)	590.04	0.17	39.76
M1 proximal (n=14)	750.61	2.3	50.9
M1 distal+M2 (n=5)	840.82	0.23	34.9
Basilar (n=5)	727.85	0.87	38.1
ASPECTS	r= 0.27<br>p= 0.15<br>n=28	r= -0.15<br>p= 0.44<br>n=28	r= 0.28<br>p= 0.13<br>n=28
Recanalization time (min)	-0.29<br>p= 0.07<br>n= 37	-0.26<br>P= 0.12<br>n= 37	r= -0.31<br>p= 0.06<br>n= 37
No. of retriever maneuvers	r= 0.05<br>p= 0.76<br>n= 37	r= -0.28<br>p= 0.1<br>n= 37	r= -0.05<br>p= 0.78<br>n= 37
NIHSS admission	r= 0.27<br>p= 0.09<br>n= 37	r= 0.18<br>p= 0.27<br>n= 37	r= 0.08<br>p= 0.6<br>n= 37
NIHSS dismissal	r= 0.29<br>p= 0.08<br>n= 37	r= -0.07<br>p= 0.96<br>n= 37	r= -0.08<br>p= 0.6<br>n= 37

Abbreviations: ICA-T = Internal carotid artery; M = Mid Cerebral Artery, ASPECTS = Alberta Stroke Program Early CT score; NIHSS = National Institutes of health Stroke Scale.
